# Endoscopic ultrasound molecular evaluation of pancreatic cancer trial to profile molecular landscape of inoperable pancreatic ductal adenocarcinoma

**DOI:** 10.1055/a-2733-1068

**Published:** 2026-01-13

**Authors:** Owen McKay, Joanne Lundy, Sally Bell, Phil Ha, Hugh Gao, Brendan Jenkins, Chamkaushalya Bulathsinghalage, Michael Swan, Simon Hew, Belinda Lee, Pranav Dorwal, Manoop S Bhutani, Vivek Rathi, Sean Grimmond, Andrew Perry, Trevor Wilson, Andrew Strickland, John Zalcberg, Daniel Croagh

**Affiliations:** 1 2538 Gastroenterology and Hepatology, Monash Health, Melbourne, Australia; 2 2538 Upper Gastrointestinal Surgery, Monash Health, Clayton, Australia; 3 5644 Medical Oncology, Peninsula Health, Frankston, Australia; 4 518514 Cancer and Immune Signalling, Hudson Institute of Medical Research, Clayton, Australia; 5 2541 Faculty of Medicine, Nursing and Health Sciences, Department of Surgery, Monash University, Melbourne, Australia; 6 5644 Gastroenterology & Hepatology, Peninsula Health, Frankston, Australia; 7 695511 South Australian ImmunoGENomics Cancer Institute (SAiGENCI), Adelaide University, Adelaide, Australia; 8 2538 Gastroenterology & Hepatology, Monash Health, Clayton, Australia; 9 3085 Medical Oncology, Peter MacCallum Cancer Centre, Melbourne, Australia; 10 5388 Medical Oncology, Walter and Eliza Hall Institute of Medical Research, Melbourne, Australia; 11 2538 Genomics & Anatomical Pathology, Monash Health, Clayton, Australia; 12 4002 Gastroenterology, University of Texas MD Anderson Cancer Center, Houston, United States; 13 2538 Genomics & Anatomical Pathology and Lifestrands Genomics Australia, Monash Health, Clayton, Australia; 14 3085 Collaborative Centre for Genomic Cancer Medicine, Peter MacCallum Cancer Centre, Melbourne, Australia; 15 2541 Monash Genomics & Bioinformatics Platform, Monash University, Melbourne, Australia; 16 518514 Hudson Genomics Facility, Hudson Institute of Medical Research, Clayton, Australia; 17 2538 Medical Oncology, Monash Health, Clayton, Australia; 18 5392 Medical Oncology, Alfred Health, Melbourne, Australia; 19 22457 Nursing and Health Sciences, Public Health, Faculty of Medicine, Monash University, Melbourne, Australia

**Keywords:** Endoscopic ultrasonography, Pancreas, Tissue diagnosis, Fine-needle aspiration/biopsy

## Abstract

**Background and study aims:**

Pancreatic ductal adenocarcinoma (PDAC) is a poor prognostic malignancy. Comprehensive genomic profiling (CGP) has improved outcomes in many cancers, but widespread uptake in PDAC remains elusive. This study investigated the feasibility of using endoscopic ultrasound with fine-needle biopsy (EUS-FNB) for CGP in advanced PDAC.

**Patients and methods (experimental design):**

A multicenter prospective cohort study was conducted to assess the feasibility of using DNA and RNA extracted from fresh frozen or archival formalin-fixed paraffin-embedded (FFPE) EUS-FNB for CGP on advanced PDAC using the TSO-500 gene panel testing. Results of the CGP were reviewed at a molecular tumor board (MTB) and subsequent treatment recommendations were forwarded to the referring clinicians.

**Results:**

CGP was successful in 129 of 143 patients (90%) enrolled between May 2020 to September 2023. Fresh frozen EUS-FNB provided suitable genetic material for CGP in 123 of 133 patients (92%). Conversely, CGP was successful on FFPE biopsy blocks from only six of 16 patients (38%). Fifty-two of 143 patients (36%) had a potentially targetable mutation detected, and eight of these patients (6%) were treated with targeted therapy based on their EUS-FNB-derived molecular profile. Patients who received personalized therapy had a significant ( *P* < 0.0001) increase in survival versus standard or no therapy at 12 and 36 months. Median patient survival on standard therapy was 9.47 months versus > 18 months for personalized therapy.

**Conclusions:**

This real-world study confirms the feasibility and utility of CGP using EUS-FNB in advanced PDAC. It illustrates the importance of timely access to personalized therapy informed by CGP, which can impact the treatment pathway and improve survival outcomes.

## Introduction

Pancreatic ductal adenocarcinoma (PDAC) is projected to become the second leading cause of cancer death overtaking colorectal cancer by 2026 1 . Median 5-year survival for all stages of pancreatic cancer remains low at 11.5% 2 . Endoscopic ultrasound-guided fine-needle biopsy (EUS-FNB) is typically used to establish the diagnosis and has a sensitivity of 85% to 89% and specificity of 96% to 100% in three meta-analyses 3 .

Germline testing is recommended in all PDAC patients, and somatic molecular testing recommended for those with locally advanced or metastatic disease by the National Comprehensive Cancer Network (NCCN), American Society of Clinical Oncology (ASCO) and the European Society for Medical Oncology (ESMO) 4 5 6 7 . Widespread uptake of comprehensive genomic profiling (CGP) into clinical practice has proven challenging due to both patient factors (poor performance status, narrow therapeutic window) and practice factors including poor quality biopsy samples, delays in processing tissue, and reliance on surgical specimens, thereby excluding the majority of patients 8 . CGP is not routinely available or subsidized in Australia but recent development of the Cancer Screening Program (CaSP) initiative has facilitated potential access to funded CGP 9 .

Historically, CGP in PDAC relied on DNA extracted from surgical resection specimens or via percutaneous biopsy 10 . Use of EUS samples for molecular profiling has been less widely examined. A cohort analysis evaluating DNA yield from EUS-guided biopsies using 19G or 22G needles found that sufficient DNA was obtained in 93% of patients, with more than DNA material obtained 11 .

We have reported on snap-frozen EUS-derived biopsies (EUS-FNB) as a feasible tissue source for a transcriptome derived genetic signature in combination with tissue *KRAS* mutational analysis to improve speed and accuracy of diagnosis and to guide targeted therapy 12 . In this retrospective cohort of 308 patients, EUS-fine-needle aspiration cytology was 78.6%. *KRAS* mutation analysis and the custom transcriptomic signature improved upon diagnostic accuracy of standard cytology to 91.6% which would suggest a role for such a transcriptome-derived genetic signature in conjunction with *KRAS* mutational analysis. A subsequent successful pilot study evaluating the role for EUS-FNB samples to guide precision medicine in pancreatic cancer in *KRAS* wild-type tumors was completed in 2021 13 . EUS-guided samples were used to procure tumor tissue and validated the possible role for EUS-FNB for mutational screening and patient-derived xenografts (PDXs) to evaluate personalized anti-epidermal growth factor receptor therapy in *KRAS* wild-type patients.

This study aimed to assess feasibility and utility of routine CGP from EUS-FNB samples to inform precision therapy in patients with advanced PDAC, to determine the proportion of patients that received personalized therapy based on their CGP results, and to compare survival of these patients with those that received standard therapy.

## Patients and methods

A prospective multicenter cohort study was designed with the goal of recruiting up to 150 patients in 3 years. Significant financial support from PanKind Cancer Foundation and the Victorian Cancer Association enabled the completion of this study. Patients enrolled in the study met all the inclusion criteria summarized in Table 1 .

**Table:** **Table 1** Study criteria.

**Inclusion criteria**	**Exclusion criteria**
Patients 18 years or older with cytologically proven PDAC by EUS, or a clinical diagnosis of PDAC made using “suspicious” cytology in conjunction with supporting biochemical and radiological data* Metastatic, locally advanced or recurrent disease ^†^ ECOG performance status 0–2 Life expectancy of at least 3 months from time of screen (judged by screening investigator) Clinically deemed suitable for chemotherapy Able to provide signed informed consent	Patients with operable or borderline resectable PDAC ^‡^ Patients with pancreatic neuroendocrine tumors (NETs) Evidence of systemic disease (cardiovascular, respiratory, renal, hepatic) that would preclude systemic therapy. Serious medical or psychiatric conditions that might compromise protocol-based management as judged by the study investigator Life expectancy < 3 months
*Defined by local multidisciplinary team review. ^†^ Those with recurrent disease with suitable lesion accessible for EUS biopsy ^‡^ Previous diagnosis of PDAC or treatment (systemic, radiotherapy or surgery did not preclude patients from being enrolled as long as the treatment intent at enrolment was non-curative (thereby not meeting exclusion criteria for operable disease). ECOG, Eastern Cooperative Oncology Group; EUS, endoscopic ultrasound; NET, neuroendocrine tumor; PDAC, pancreatic ductal adenocarcinoma. Surgery currently remains the mainstay of curative intent therapy for patients with borderline resectable or resectable PDAC with no clear role established for molecularly targeted therapies in this group, and they were they were therefore not included in this study population. In such patients, there is also a theoretical (exceptionally rare) risk of seeding and including them may potentially compromise their resectability for those with pancreatic body and tail lesions that require a transgastric approach for biopsy.	

### Ethics oversight

The study was approved by the Human Research Ethics Committee (HREC), Monash Health and registered with the Australian New Zealand Clinical Trials Registry (ACTRN12620000762954). It was conducted in accordance with Good Clinical Practice, and the ethical principles in the Declaration of Helsinki.

### Tissue acquisition

Tissue acquisition was completed by expert endoscopists in EUS and the patients received deep sedation in the form of either “twilight” sedation with propofol or general anesthesia. No patients were conscious for the procedure, allowing maximal tissue acquisition.

A single additional pass at the time of diagnostic EUS was completed and placed into 1 mL of saline, frozen and stored at -80 ^o^ C. If fresh frozen tissue was not available, then CGP was performed on genetic material extracted from archival formalin fixed paraffin embedded (FFPE) tissue scrolls. Fresh frozen tissue was prioritized due to the concern of formalin/paraffin embedding potentially compromising genetic material and thereby impacting genetic profiling/analysis 14 . Patients were offered further EUS-FNB procedures for the purpose of obtaining fresh frozen tissue if the extracted DNA/RNA was insufficient for detailed analysis. The number of passes for routine diagnostic cytology purposes, needle choice, use of rapid onsite cytological evaluation (ROSE), and macroscopic slide evaluation were at the discretion of the endoscopist. All endoscopists used core needles with the Franseen tip design (either the 22G Acquire Boston Scientific needle or 20G Procore from Cook).

### Molecular profiling

Total RNA and DNA were extracted from fresh frozen samples using the AllPrep DNA/RNA Universal Kit (QIAGEN). If no fresh frozen tissue available, gDNA was extracted from FFPE tissue scrolls made from diagnostic EUS-FNB. Isolation of gDNA was prioritized for FFPE samples and performed on 5 × 10 micron-thick sections using the ReliaPrep FFPE gDNA Miniprep System (Promega). The quality and quantity of DNA and RNA was determined by Nanodrop Spectrophotometer and Qubit reagents (Thermo Fisher Scientific, Waltham MA), and Tapestation reagents (Integrated Sciences). CGP was performed using the TruSight 500 assay (TSO-500, Illumina). The TSO-500 is a pan-cancer genomic assay which uses hybrid capture chemistry to target both 523 DNA genes and 55 RNA genes, microsatellite instability (MSI) and tumor mutation burden (TMB) 14 .

### Molecular tumor board

TSO-500 gene panel was processed through the Pierian Dx platform and variant curation completed for each patient outlining the TMB, microsatellite status, clinically relevant variants and variants of unknown significance completed. Reports were reviewed at a local multidisciplinary molecular tumor board comprising oncologists, molecular pathologists, pancreatic surgeons, gastroenterologists, interventional endoscopists, scientists, and trial staff. The outcome of this was documented and the report sent to referring specialist.

### Definition of successful CGP

CGP success was defined as detection of at least one relevant common oncogene (such as a KRAS mutation) with a minimum mutant allele frequency (MAF) of at least 1%. In addition, CGP was considered successful in *KRAS* wild-type cases when other canonical mutations were identified such as *BRAF* and *GNAS* 15 .

### Definition of potentially targetable molecular alterations

“Targetable” is a dynamic concept with the evolving molecular understanding of PDAC and subsequent development of therapies to selectively target molecular alterations identified in these patients. We defined potentially targetable molecular mutations as those with an established targeted therapy, such as poly ADP ribose polymerase (PARP) inhibitors for *BRCA* -mutant tumors 16 or mutations with active or relevant clinical trials available in the state with target specific therapies available. These findings and targeted therapy/clinical trial options were documented in the setting of a formal molecular tumor board (MTB) with multidisciplinary input from relevant specialties.

### Follow up

Patients were followed for up to 36 months after recruitment by the study investigators or until deceased, and treatment and survival data reported here at the time of data cut-off on November 31, 2024. Patients and their relevant next of kin contacts were seen face to face or via telehealth review with study investigators.

### Statistical considerations

This study aimed to enroll up to 150 patients over 3 years with the goal of achieving 100 adequate DNA/RNA extractions for CGP. The rationale for this number was based on an expected 33% RNA extraction failure rate identified in previous work 12 , and the expectation of seeing approximately 50 potentially suitable patients each year. Mean and standard deviation are reported in normally distributed continuous data. For skewed distributions, we reported the median and interquartile range (IQR). For exponential distributions, data log transformed prior to subsequent analysis. The t-test was used to compare means of normally distributed data between groups, while the nonparametric Mann-Whitney (rank sum) test was used to compare distributions of skewed data. To analyze the outcome of survival, we utilized Kaplan Meier analysis with log rank testing to assess difference between groups on survival. All analyses were conducted with STATA version 18 (StataCorp, TX, USA) and a *P* < 0.05 was considered statistically significant.

## Results

### Overall CGP success

Oncogenic variants were detected in 129 of 143 (90%) at a MAF > 1%. CGP was not possible in 14 patients due to inability to obtain sufficient quantity and quality of DNA, or if no patho-diagnostic mutations with a MAF > 1% were found on the TSO-500 gene panel. A total of 111 patients (78%) had one procedure, 15 patients (11%) required two procedures, and three patients (2%) required three procedures for successful CGP. Eighteen of 140 patients had systemic therapy prior to the EUS-FNB to obtain tissue for genomic profiling. Of note, there was no difference in overall rate of success of CGP for these patients compared with those who had not had prior therapy. Systemic therapy can potentially alter the MSI status in these tumors and may impact interpretation of molecular findings in these patients. One targetable mutation ( *KRAS* G12C) was detected on a clinically validated *KRAS* StripAssay (ViennaLab Diagnostics) and not on the subsequent TSO-500 gene panel testing for CGP and was considered a failed CGP 8 . Presumably the diagnostic material had been exhausted when the tissue block had been accessed for the *KRAS* StripAssay.

**Fig. 1:**
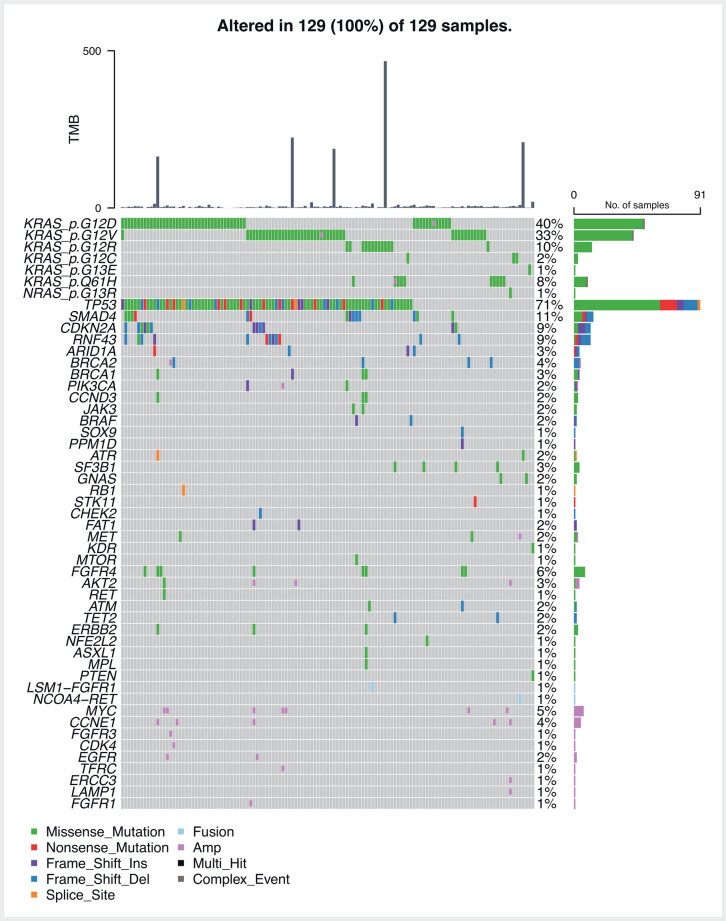
Oncoplot – pathogenic variant mutations detected on TSO-500 panel.

DNA quality and quantity was higher in the fresh frozen cohort and no RNA was extracted from the FFPE (DNA extraction was prioritized). The statistical detail around these two types of specimens is included in a supplementary file ( **Supplementary Table 1** ). Six patients with FFPE samples were included and only six of 16 (38%) had suitable material for successful CGP. Six of 10 of the failed FFPE patients were offered and underwent a repeat EUS-FNB for fresh tissue acquisition for re-attempt at CGP; five had successful CGP from this additional biopsy. Overall, 52 of 143 patients (36%) were found to have potentially actionable molecular alterations – summarized in Table 2 . The variety of pathogenic variant mutations detected from the TSO-500 genel panel testing for the patients in this study are illustrated in Fig. 1 .

**Table:** **Table 2** Targetable mutations and frequency.

KRAS G12C	3
KRAS G12D***	2
PIK3CA	4
RNF43	11
ERBB2 (HER-2)	3
ATM	2
ATR	2
CHEK2	1
MET amp	2
RET-NCOA4 fusion	1
BRCA2	4
BRCA1	4
TMB	11
**Total**	**52/143 (36%)**
*** Overall, 51/143 (36%) were found to have KRAS G12D mutations which we now know to be potentially targetable at the time of publication of this article. However, we have listed two patients as actionable at the time of enrolment in the trial due to direct access to clinical trials that were G12D specific.	

Pathogenic KRAS mutations were detected in 122 patients, whereas seven patients were KRAS wild-type but had other patho-diagnostic alterations (including BRAF, GNAS, RET fusion, and MET amplification). MAF for pathogenic KRAS variants ranged from 0.8% to 89.6% with a median of 13.5%. The KRAS MAF percentage was > 1% in 121 of 122 of these patients. The one patient with a low KRAS MAF of 0.8% had a TP53 mutation with MAF > 1%. Ninety patients had a TP53 mutation, 89 of 90 of these had a TP53 MAF > 1% (median 19.3%) with the one patient having a low TP53 MAF of 0.8% also having another patho-diagnostic mutation detected resulting in successful CGP of this sample. The mean number of common mutations per patient with successful CGP was 1.73 (±0.85). Fifty-one of 143 patients (36%) in our study were found to have KRAS G12D mutations. Clinical trials evaluating KRAS G12D inhibitors opened towards the end of recruitment for patients in this study. Patients with this mutation are now considered potentially targetable on the basis of their molecular mutation. Only two of the patients with G12D mutations were considered potentially targetable as G12D specific trials opened in time to allow their enrolment into one of these trials.

Median TMB for this cohort of patients was 3.1 Mut/Mb (Range 0–466.9 Mut/Mb). As expected, the majority of patients (118/129) had a low TMB < 10 Mut/Mb, whereas six patients had high TMB (10–100 Mut/Mb), and five patients had hypermutated tumors with markedly elevated TMB > 100 Mut/Mb. Interestingly, all processed samples including the hypermutated TMB patients were microsatellite stable. Only four of 11 samples had immunohistochemistry for MMR proteins and in these cases the results correlated with molecular findings of microsatellite stability. Eleven of 129 patients (9%) with a TMB > 10 Mut/Mb was higher than expected for this patient cohort. There was no correlation between the KRAS (or TP53) MAF and TMB levels, suggesting that low tumor cellularity was not the explanation for the higher-than-expected frequency of high TMB tumors.

Eight patients (6%) were started on targeted therapy as a result of the molecular findings. Three patients with hypermutated tumors have commenced immunotherapy (with treatment durations of > 3, > 2 and ~1 year). One of these patients also had a *BRCA* mutation and received a PARP inhibitor 17 . Two patients with *KRAS* G12C mutations have commenced on targeted therapy (with treatment durations of greater than 2 years each), two patients with *BRCA* mutations commenced PARP inhibitors and platinum-based therapies, one patient was given *KRAS* G12D selective inhibitors in a clinical trial but had progression of disease before being switched to an alternate therapy. Five of the eight patients who were started on personalized therapy were still alive at the conclusion of this study, preventing a formal calculation of median survival for this cohort. Based on current time from biopsy, though, this median survival in the personalized therapy cohort was over 21 months (compared with 9.46 months in the standard therapy cohort).Three patients have had a complete response to therapy (1 x patient with BRCA1 / high TMB, 2 x patients with G12C mutations) including one patient with a sustained complete response for over 48 months 17 .

**Fig. 2:**
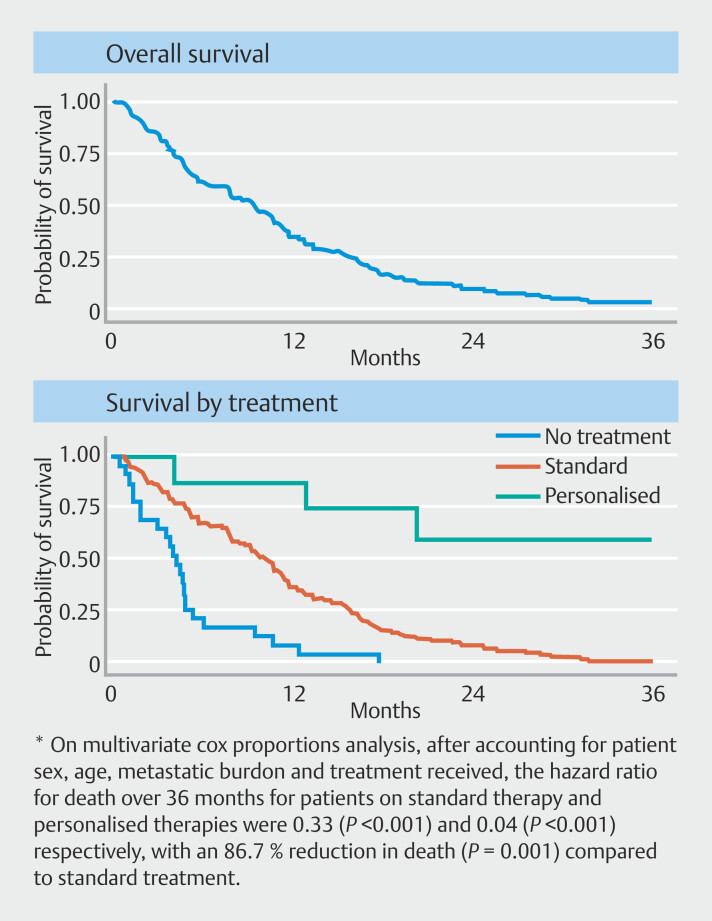
Overall survival and Kaplan-Meier curves of survival based on treatment type.

**Fig. 3:**
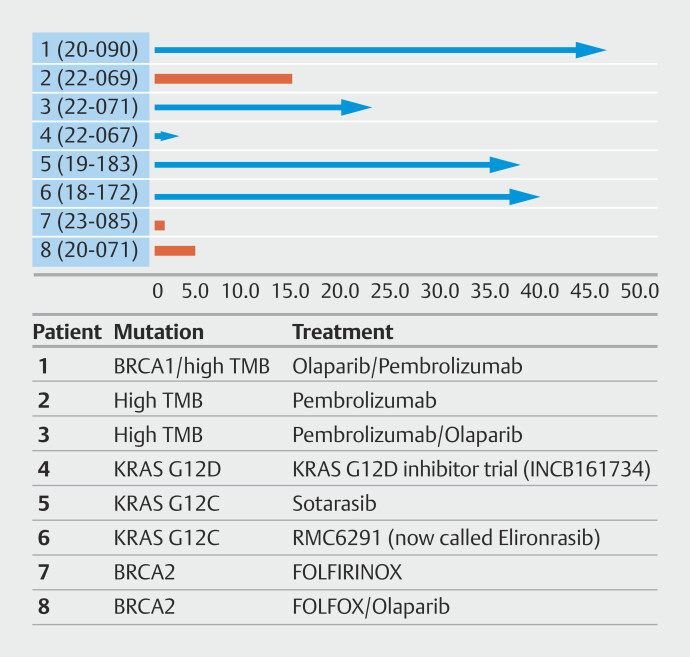
Personalized therapy.

Overall survival at 12 and 36 months were 35.7% and 6.3%, respectively, for patients enrolled in this study. Presence of a targetable mutation did not change median survival (11.0 months IQR 4–16.4 vs 7.8 months IQR 3.8–15.5). On log rank testing, there was a difference in survival between patients who received no treatment, standard treatment and second line sequence driven personalized treatment. This was significant for follow-up at 12 months ( *P* < 0.0001) and 36 months ( *P* < 0.0001). Fig. 2 is a summary of the survival of each of the cohorts based on their treatment categories:

The patients that were started on personalized therapy and their survival in months are illustrated in Fig. 3 (time on treatment for patients with personalized therapy by molecular alterations detected on CGP in this study [x axis = months alive]).

**Fig. 4:**
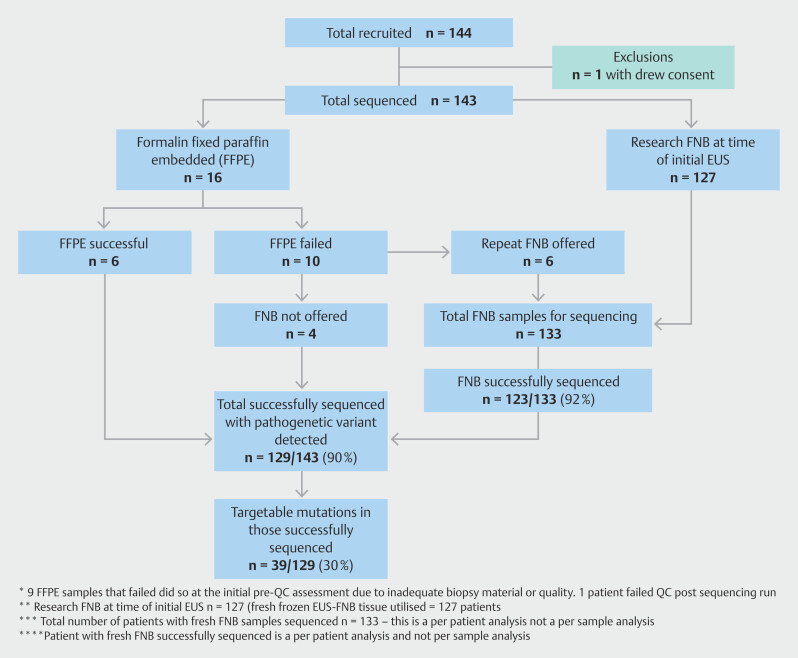
Flowchart of recruited patients in EU ME PC 1 study.

Fig. 4 is a flowchart of the recruited patients in the trial and the overall patient demographics from participants enrolled in the study are summarized in Table 3 .

**Table:** **Table 3** Patient demographics in the EU ME PC 1 study (n = 143).

**Sex**	
Female	62 (43.4%)
Male	81 (56.6%)
**Age (mean)**	67.4 (29.6–82.6)
**CA19–9 (median)**	298 (73–1744)
**Staging (III or IV)**	
III	42 (29.4%)
IV	101 (70.6%)
**Number of metastatic sites**	
0	42 (29.4%)
1	48 (33.6%)
2	43 (30.0%)
3	10 (7.0%)
Previous treatment*	
Systemic	18 (12.9%)
Radiotherapy	1 (0.7%)
Surgery	3 (2.1%)
*1 x patient withdrew prior to sequencing This cohort of patients (21 patients) who had undergone previous treatment were enrolled in the trial and samples obtained were taken after these treatments, which may have impacted the CGP result.	

## Discussion

Widespread uptake of CGP in PDAC remains problematic despite it being recommended as standard of care. EUS-FNB holds significant appeal to facilitate CGP but there is limited existing literature on using EUS biopsies for CGP in PDAC with variable results 18 19 .

Some previous larger studies have relied FFPE surgical resection specimens or percutaneous biopsies (IMPACT and COMPASS trials) 20 21 . Accordingly, we conducted a study to demonstrate feasibility and utility of EUS-FNB to enable routine CGP for these patients. EUS-FNB samples facilitated successful CGP in 92% of patients in this study, which is consistent or improved on the rates seen in a systematic review and meta-analysis from 2024 22 . Thirty-six percent had potentially targetable mutations identified and 6% were started on personalized therapy on the basis of CGP results. These results from our prospective study are encouraging when compared with other studies examining the role of EUS biopsies for CGP, including a recent retrospective study from the United States which noted 71.4% of FNB samples and only 32.1% FFPE sample being suitable for CGP 18 . Fresh frozen tissue allows preservation of DNA and RNA integrity, thereby by reducing the potential fragmentation often seen when using FFPE samples.

Successful CGP was defined in our study when two criteria were met: first, adequate quantity and quality of DNA (+/- RNA) and second, detection of relevant patho-diagnostic mutations with a MAF of at least 1%. This threshold, which is lower than the standard threshold of 5% MAF, was selected given that typical activating oncogenic mutations (e.g. mutant KRAS) are well defined in pancreatic cancer and EUS biopsies are likely to have a lower MAF than other sampling techniques due to presence of non-tumor cells within the biopsy (e.g. duodenal and gastric wall contaminants). Tumor cellularity was impossible to assess directly from these samples because the entire biopsy was utilized for DNA and RNA extraction. As a surrogate marker of tumor cellularity, we compared the MAF of *KRAS* and *TP53* mutations using a Spearman’s correlation and confirmed there was a strong correlation between the *KRAS* MAF and *TP53* MAF (r 89 = 0.79, *P* < 0.00001) suggesting they are reasonable surrogate markers for tumor cellularity here.

The rate of successful CGP in our cohort mirrored the rate of success of the underlying EUS-FNB cytology result for our patients: the diagnostic cytology correlated with successful CGP in 94% of cases. Thirteen patients were included in the study in whom the paired biopsies that were sent for cytological assessment were non-diagnostic. These patients were included after additional biopsy procedures provided an adequate diagnosis. Ten of 13 (77%) of these participants had successful CGP despite the non-diagnostic cytology result. Interestingly, there was no statistical difference in the MAF in patients who had diagnostic cytology results obtained at the same procedure compared to those with non-diagnostic cytology, suggesting that low tumor cellularity within the biopsy was not the likely cause of the false-negative cytology result.

Despite improved needle technology and procedure technique, a confirmatory diagnostic cytology result is not always possible 10 23 . A 2023 nationwide Dutch study evaluated the rate of adequate sample and sensitivity for malignancy (SFM) in patients who underwent EUS-FNB prior to surgical resection of PDAC. This study illustrated the a highly variable SFM confirmation (20%-77%) and significant practice variation across different centers 24 . The high rate of identification of patho-diagnostic mutations in patients with non-diagnostic cytology suggests that there may be a role for a “molecular diagnosis” of pancreatic cancer when conventional cytology fails.

A previous pan-cancer study revealed that PDAC typically has a lower level TMB compared with other types of cancer 25 . Eleven patients in this study had a high TMB defined as > 10 Mut/Mb. These results were higher than expected and we considered whether the nature of the EUS biopsy might explain this. However, there was a lack of correlation between the MAF and the TMB, suggesting that low tumor cellularity is unlikely an explanation for these higher TMB results. They could represent a statistical anomaly or potentially been underestimated in previous studies.

The optimal strategy for molecular characterization of PDAC biopsy material remains unclear 14 . The TSO-500 gene panel platform was utilized in this study and the majority of mutations detected were found from extracted DNA material. Only two RNA fusion mutations of clinical relevance (LSM1-FGFR1 and NCOA4-RET) were detected, despite adequate RNA in 80% of the patients who had fresh frozen tissue available. In a recent randomized trial, Bang et al were able to obtain adequate RNA material for CGP in 31 of 33 patients (94%) from FFPE material obtained from either two or three passes of an FNB needle 23 . Despite, their success with RNA extraction from FFPE, Bang et al. detected only one patient with a somatic oncogene RNA fusion mutation (LDAH-ETV1) which was classified as clinically relevant, inferring a likelihood of failure to respond to aggressive chemotherapy 23 . No clinically relevant RNA mutations were detected in our study using the TSO-500 gene panel. Current molecular targets for precision therapies in PDAC have largely focused on DNA alterations, rather than RNA alterations 26 27 . However, there is increasing awareness of RNA-based alterations playing a role in the progression of pancreatic cancer including, noncoding RNAs, splicing and transcriptomic mutations and evolving research may identify RNA targets that may be relevant to diagnosis, prognosis and potentially new therapeutic targets in the future 26 28 .

Recent studies have reported on results of molecular screening of patients and implementation of targeted therapies in advanced PDAC 16 21 29 30 . The Know Your Tumor study reported on 1856 pancreatic patients referred for molecular testing, and noted that those the 46 patients who received matched therapies based on their CGP findings had significantly longer survival (2.58 years) compared to those who received unmatched therapies (1.32 years) 30 . Similarly, a prospective study of 895 patients managed through a MTB in the United Kingdom reported that although targetable mutations were detected in 20% of patients, only 7% of these patients actually received the recommended matched therapies 29 . A single-center experience from Munich reported on 165 patients with PDAC reviewed at MTB with 25% having potentially targetable mutations identified but only 3.2% of patients were able to start on targeted therapy. Our study had a higher population with potentially targetable mutations (52/143 or 36%) but only a small subset of patients (8 patients or 6%) started on targeted therapies, due to limited targeted drug availability, or disease progression.

Our study illustrates the challenges of real-world implementation of CGP in PDAC. These patients have a narrow clinical therapeutic window, emphasizing the need for an early, streamlined approach to CGP to allow them the opportunity to have potentially targetable therapy. Median time to MTB review from enrollment in the study was 76.6 days (IQR 57–113.5), which could have impacted the ability of some participants to access targeted therapy. There are several explanations for the lengthy delay between enrollment and MTB review for our patients. First, to reduce cost, we needed to batch sequence the patients, running eight samples at a time. The cost of sequencing using the TSO-500 gene panel was covered for participants in this study with a trial grant (approximately $2000AUD per patient). These data are needed to compel funders to support this approach in the future. The establishment of subsidized programs such as CaSP holds great appeal if sequencing results can be returned expeditiously to potentially impact patient treatment options and outcomes 9 .

Streamlining of CGP requires the infrastructure for variant curation and MTB review with emerging data showing that MTBs guided treatment for patients with advanced solid tumors to impact survival outcomes 31 . CGP generates large amounts of complex data, and requires both a molecular pathologist to curate the mutational profile and identify pathological mutations, and experienced oncologists with knowledge of available targeted therapies to achieve clinical benefits for these patients 14 29 .

This study relied on the preestablished infrastructure of a cancer biobank to store the fresh EUS-FNB sample. Such infrastructure is not widely available, potentially impacting the generalizability of this approach in the community. Other pathways should be considered, including evaluating the role of residual liquid (the supernatant) generated during routine processing of EUS-FNB for cytological diagnosis. The supernatant has been demonstrated to contain genetic material with potential clinical utility 32 . Although this method necessitates streamlined processing into an appropriate media rather than formalin, it offers the advantage of bypassing the need for a dedicate biobank infrastructure.

Finally, targeted therapies are not always easily accessible in clinical practice. Next generation sequencing technology and bioinformatics have driven the discovery of driver mutations and altered pathways in PDAC that have shown promise in preclinical and clinical studies such as sotorasib for *KRAS* G12C-mutated PDAC patients 33 . Evolution of repurposed or novel therapies for previously non-targetable mutations provides hope for the future 34 .

## Conclusions

This prospective multicenter cohort study demonstrates the feasibility and utility of EUS-FNB for tissue acquisition for CGP. This study is one of the first to validate use of the TSO-500 panel in this setting and highlights the importance of streamlined tissue processing, accessible variant curation, and MTB opinion in delivering results that influence treatment. There is an unmet clinical need for integration of targeted therapeutic options for patients with advanced PDAC. Despite the well-documented challenges, the value of molecular profiling to guide treatment selection for patients with PDAC is clear, with a marked survival advantage seen in our exploratory analysis for patients who can commence targeted therapy. As more targeted therapies such as *KRAS* G12D inhibitors become available, access to therapy should increase and generate further improvements in outcome for patients with inoperable PDAC.

## Supplementary Material

Supplementary Material Zusatzmaterial
