# Supplementary material for: Endoscopic ultrasound molecular evaluation of pancreatic cancer trial to profile molecular landscape of inoperable pancreatic ductal adenocarcinoma
Source: Endosc Int Open. 2026 Jan 13;14:a27331068. doi: 10.1055/a-2733-1068 (PMC12817188; doi:10.1055/a-2733-1068)
Supplement: Supplementary file 1 — Supplementary Material [file 10-1055-a-2733-1068_27438250.pdf]

Supplementary Table 1 DNA/RNA quantity and quality from fresh frozen vs. FFPE samples.

	**Successful FNB (n = 123)**	**Successful FFPE (n = 6)**	**(*P* value Mann-Whitney u)**
DNA QuBit median ng/uL (IQR)	38.6 (IQR 10.4-97.3)	7 (IQR 4-9.6)	*P* = 0.0003
DIN median (IQR)	7.1 (IQR 6.5-7.6)	2.3 (IQR 2.2-2.4)	*P* = 0.0001
RNA QuBit median ng/uL (IQR)	48.4 (IQR 17.9-94)	No sample for RNA extraction (prioritized for DNA assessment)	N/A
RIN median (IQR)	2.7 (IQR 2.5-3.4)	No sample for RNA extraction (prioritized for DNA analysis)	N/A

FFPE, formalin-fixed paraffin-embedded; FNB, fine-needle biopsy; IQR, interquartile range.
